# Salivary adenoid cystic carcinoma with an early phase of high-grade transformation: case report with an immunohistochemical analysis

**DOI:** 10.1186/1746-1596-8-113

**Published:** 2013-07-02

**Authors:** Kimihide Kusafuka, Tomoko Miki, Takashi Nakajima

**Affiliations:** 1 Pathology Division, Shizuoka Cancer Center, 1007 Shimonagakubo, Nagaizumi-cho, Sunto-gun, Shizuoka, 411-8777, Japan

**Keywords:** High-grade Transformation, Adenoid Cystic Carcinoma, Salivary Gland, Early Phase, Immunohistochemstry

## Abstract

**Background:**

The early phase of salivary gland carcinomas with high-grade transformation (HGT) is extremely rare. We reported one case of adenoid cystic carcinoma (AdCC) with early HGT, herein.

**Case presentation:**

The patient was a 27-year-old Japanese woman who suffered from swelling of the left parotid region. Most of this tumor consisted of typical AdCC histology, whereas the central area of this tumor was composed of solid growth component by atypical cells with clear cytoplasm and marked nuclear atypia. Immunohistochemically, this area was strongly and diffusely positive for epithelial membrane antigen, p53, p16, Her-2, cyclin A and cyclin B1. The Ki-67 labeling index of this area was high, entirely different from that of AdCC area.

**Conclusion:**

Overall, this area was an early phase of AdCC-HGT. This case is the second case of early AdCC-HGT. We discuss the development of salivary gland carcinoma with HGT.

**Virtual Slides:**

http://www.diagnosticpatology.diagnomx.eu/vx/1598278104895730

## Background

The concept of dedifferentiation or high-grade transformation (HGT) in salivary gland carcinoma derived from the one of bone and soft tissue sarcomas. Cheuk *et al.* reported the adenoid cystic carcinomas (AdCC) with HGT in 1999 [1]. Ide *et al.* reported the incipient phase of AdCC-HGT [2], but the early phase of the HGT of AdCC remains unclear. We report herein one additional case showing the early phase of HGT in salivary AdCC.

## Case presentation

### Clinical history

**Figure 1:**
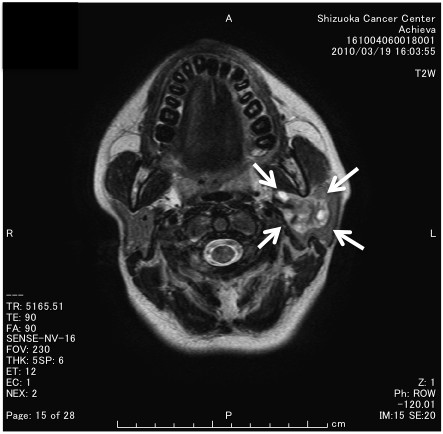
Magnetic resonance imaging (T2 weight imaging) showed an irregularly lobulated mass with irregularly low-intensity and partly high-intensity (white arrows) in the left parotid gland.

The patient was a 27-year-old Japanese woman who suffered from the swelling of the left parotid region. She had never admitted to any other hospitals for 9 years, and then, she admitted to our hospital. Computed tomography indicated a 30 × 20 × 25 mm irregular-shaped mass was seen in the left parotid gland. Magnetic resonance imaging showed an irregular-shaped mass, which showed the low intensity with T1-weight imaging and the low and partly high intensity with T2-weight imaging (Figure 1). Total parotidectomy and neck dissection were performed one month later under the clinical diagnosis of parotid gland carcinoma. Multiple lung metastases, pelvic metastasis and loco-regional recurrence were observed two months after operation. Although the palliative radiotherapy was performed, she died of disease 6 months after the operation. Autopsy was not permitted.

### Pathological findings

**Figure 2:**
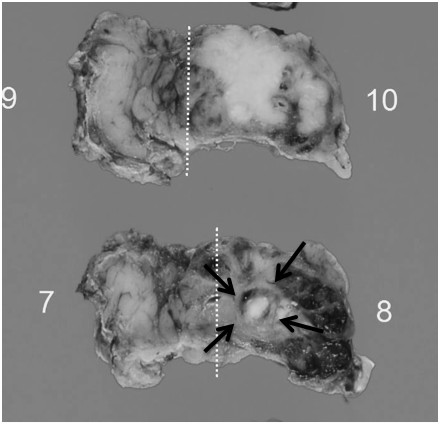
**Macroscopically, the tumor showed an ill-defined mass, and a nodular focus (arrows) in the main lesion (white dotted lines are the sectioning lines).** The nodular focus was comparable to the HGT component.

**Figure 3:**
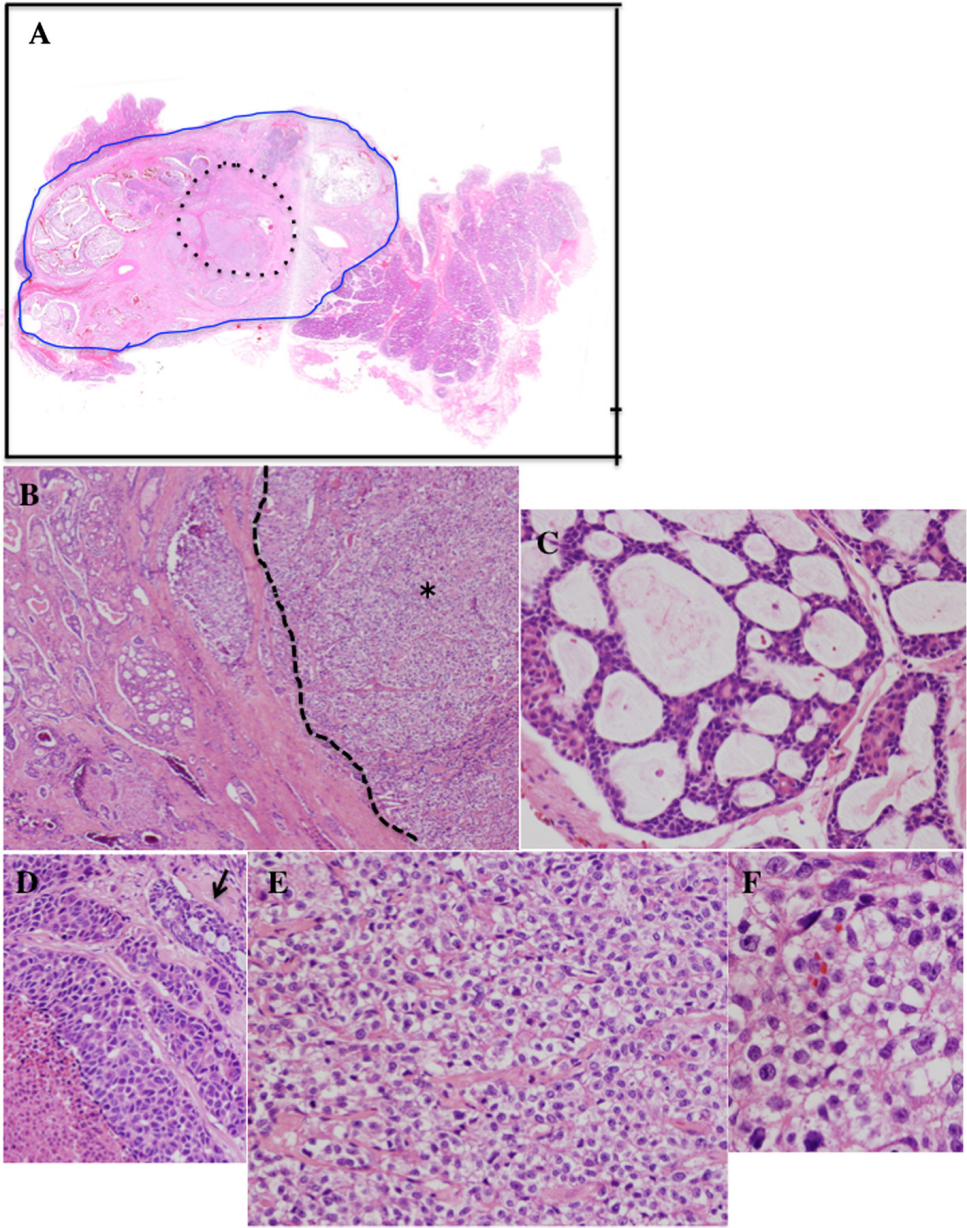
**Histological findings of the present case.** A high-grade carcinoma (black dotted line), which was different from the adenoid cystic carcinoma (AdCC) component (blue line), was seen in the central area of this tumor (**A**: whole mount H&E stain section). A focus of high-grade carcinoma (asterisk) was well demarcated in typical AdCC area and no gradual transition zone was seen (black dotted line) (**B**: H&E stain x20). Typical of AdCC showed a cribriform pattern (**C**: H&E stain x200). Solid subtype of AdCC was seen partly (arrow shows cribriform pattern within the solid subtype of AdCC.) (**D**: H&E stain x200). High-grade carcinoma showed solid and insular growth (**E**: H&E stain x200) with clear cytoplasm and marked nuclear atypia (F: H&E stain x400).

**Figure 4:**
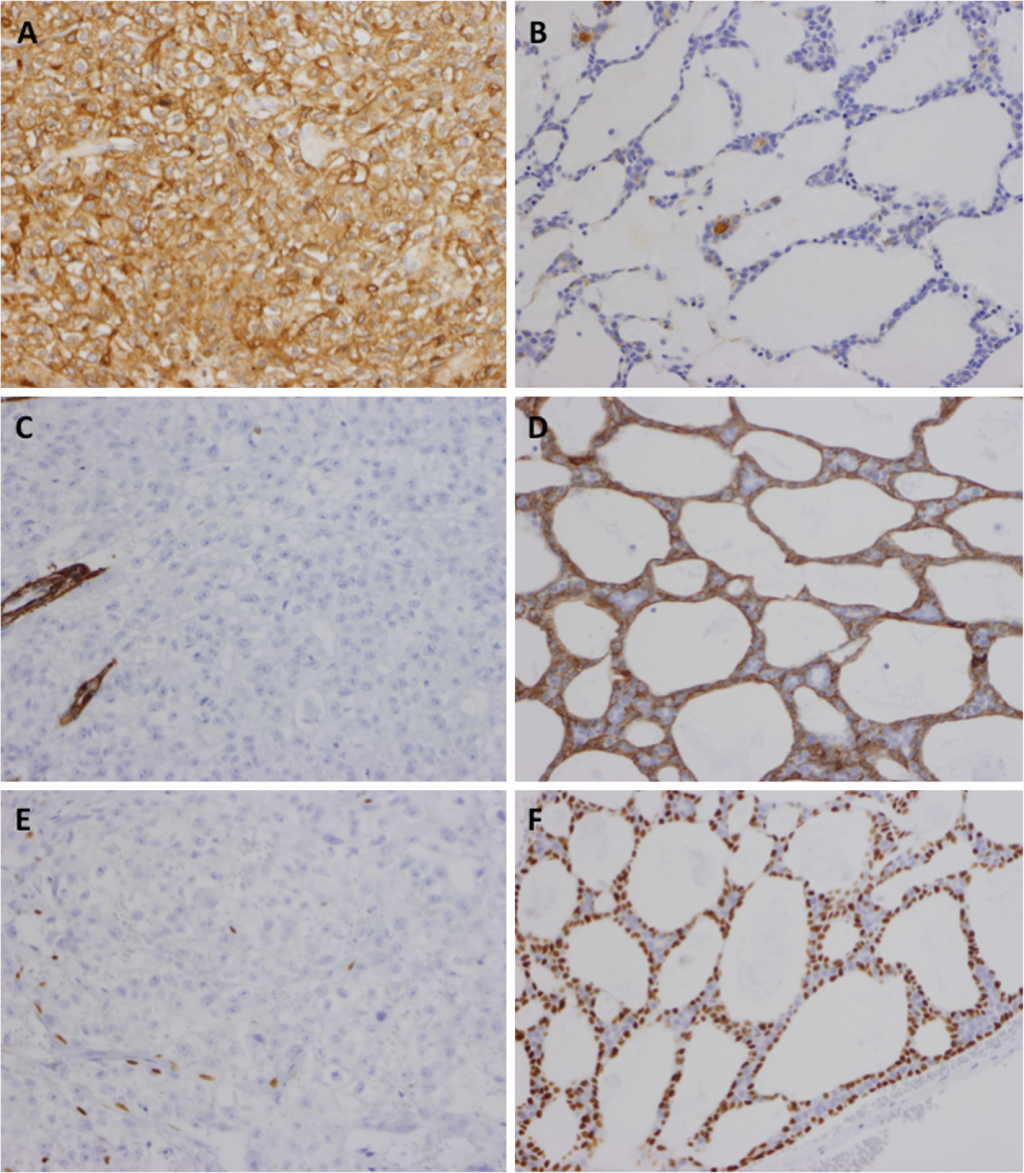
**The immuno-phenotypes of high-grade carcinoma, comparing with those of typical AdCC.** The high-grade carcinoma component showed strong and diffuse positivity for EMA **(A**: immunostaining x200), whereas the typical AdCC component showed limited positivity forthe true lumens in the cribriform growth area **(B**: immunostaining x200). The high-grade carcinoma component was negative for ASMA **(C**: immunostainng x200: the blood vessels were positive**)**, whereas the peripheral cells adjacent the pseudocysts in the AdCC area were positive for ASMA **(D**: immunostaining x200). The high-grade carcinoma component was negative for p63 **(E**: immnostaining x200: the involved myoepithelial cells were positive), whereas the peripheral cells adjacent the pseudocysts in typical AdCC area were positive for p63 **(F**: immunostaining x200**)**.

**Figure 5:**
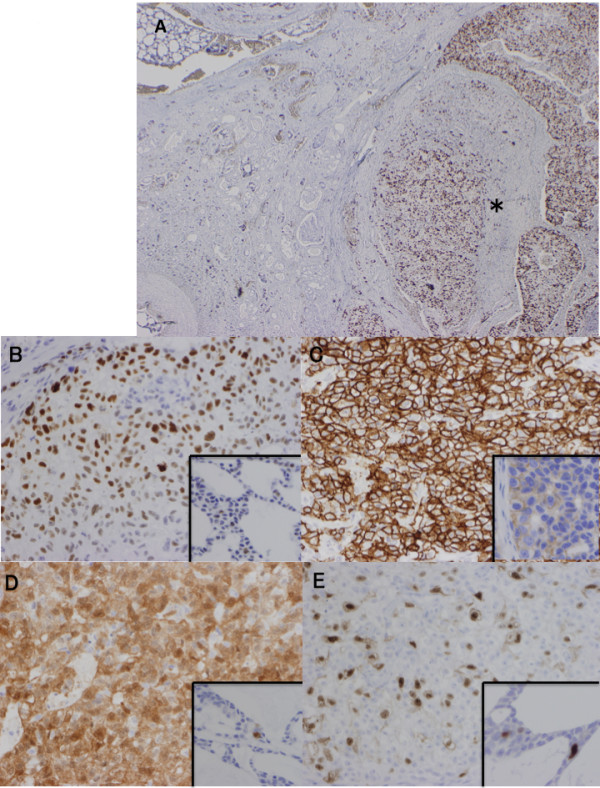
**Results of the immunohistochemical examination of high-grade carcinoma component in the present case.** Typical AdCC area showed low labeling index for Ki-67, whereas the high-grade carcinoma component (asterisk) showed a high Ki-67 labeling index **(A**: immunostaining x20**)**. The high-grade carcinoma component also showed strong and diffuse positivity for p53 **(B**: immunostaining x200: inset; weakly positive for p53 in typical AdCC**)**, Her-2 **(C**; inset; weakly positive for Her-2 in typical AdCC: immunostaining x400**)**, p16 **(D**: immunostaining x200: inset; focally positivity for p16 in typical AdCC; immunostaining x400**)**, and cyclin A **(E**; immunostaining x200: inset; focally positivity for cyclin A in typical AdCC; immunostaining x400**)**, respectively.

Macroscopically, an ill-defined grayish-white mass was observed in the left parotid gland (Figure 2). Histologically, most part of the tumor showed a cribriform growth pattern of the relatively small tumor cells, which could be diagnosed as typical AdCC (Figure 3A,B,C), whereas about 10% of the tumor was composed of the solid subtype of AdCC (grade II) (Figure 3D). In the central portion of the tumor (approximately 10% of this tumor: the size was 7 × 5 mm), an entirely different component from AdCC was observed, which consisted of solid and sheet-like growth by atypical cells with clear cytoplasm and marked nuclear atypia (Figure 3E). The tumor cells in this area, which were larger than typical AdCC cells, showed prominent nucleoli and irregular-shaped nuclei (Figure 3F). Such a component lacked ductal-myoepithelial differentiation. Although necrosis was seen, perineural invasion, and angiolymphatic invasion were not observed in “the sheet-like area”. There was no transitional zone between conventional AdCC areas and “the sheet-like area”. In AdCC component, periodic acid-Schiff (PAS)-positive lumens were seen, but “the sheet-like area” was negative for PAS and Alcian blue staining. The surgical margin was positive and the extraglandular extension was observed. Immunohistochemistry was performed on the deparaffinized tissue sections using the primary antibodies listed in Table 1 and the EnVision (DakoCytomation, Carpinteria, CA, USA) method. Immunostaining showed that the only true lumen of the typical AdCC portion was positive for epithelial membrane antigen (EMA), whereas “the sheet-like area” was diffusely positive for EMA. The AdCC area was positive for S-100 protein, p63, and cytokeratin (CK) 14 and alpha-smooth muscle actin, whereas “the sheet-like area” was negative for such antigens, except for the positivity for p63 and CK14 in the myoepithelial cells (Figure 4). Moreover, the AdCC area was weakly positive for Her-2 and the negativity for p16, whereas “the sheet-like area” revealed diffuse and strong positivity for such antigens. The AdCC area showed sporadic positivity for p53, cyclin A and cyclin B1, whereas “the sheet-like area” revealed diffuse and strong positivity for such antigens. The Ki-67 labeling index of the AdCC area and “the sheet-like area” showed 34.5% and 73.2%, respectively (Figure 5A). “The sheet-like area” indicated the overexpression of p53, p16, Her-2, cyclin A and cyclin B1 (Figure 5B,C,D,E), different from those of the typical AdCC.

**Table 1:** The antibodies used in this study and their results

	**Antibody**		**ACC**		**HGT**
**Antigens**	**Clone**	**Source**	**Cribriform**	**Solid**	
EMA	E29	DakoCytomation (Carpinteria, CA, USA)	+	+	^++^
GCDFP-15	NCL-GCDFP15	Novocastra Laboratories Ltd. (New Castle Upon Tyne, UK)	-	-	-
Her-2		DakoCytomation (Carpinteria CA)	-	1+	3+
EGFR	NCL-L-EGFR	Novocastra Laboratories Ltd. (New Castle Upon Tyne, UK)	-	-	-
AR	AR441	DakoCytomation (Carpinteria, CA, USA)	-	-	-
CK5/6	D5/16 B4	DakoCytomation (Carpinteria, CA, USA)	+	+	-(myo+)
S-100		DakoCytomation (Carpinteria, CA, USA)	+	+	-
ASMA	1A4	DakoCytomation (Carpinteria, CA, USA)	++	+	-
calponin	CALP	DakoCytomation (Carpinteria, CA, USA)	+	f+	-
CK14	LL002	Chemicon International (Temecula, CA, USA)	+	p+	-(myo+)
p63	4A4	Lab Vision (Fremont, CA, USA)	++	p+	-(myo+)
mammaglobin	304-1A5	DakoCytomation (Carpinteria, CA, USA)	-	f+	
p53	DO-7	DakoCytomation (Carpinteria, CA, USA)	f+	p+	++
cyclin A	NCL-CYCLN A	Novocastra Laboratories Ltd. (New Castle Upon Tyne, UK)	10.3%	17.3%	34.5%
cyclin B1	NCL-CYCLN B1	Novocastra Laboratories Ltd. (New Castle Upon Tyne, UK)	0.5%	12.2%	50.3%
cyclin D1	DSC-6	DakoCytomation (Carpinteria CA, USA)	-	1.1%	-
cylcin E	NCL-CYCLN E	Novocastra Laboratories Ltd. (New Castle Upon Tyne, UK)	-	-	-
p16	G175-405	BD Biosciences (Franklin Lakes, NJ, USA)	f+	p+	++
MDM2	SMP14	Themo Fisher Scientific (Cheshire, UK)	-	-	-
Ki-67L.I.	MIB-1	DakoCytomation (Carpinteria, CA, USA)	34.5%	49.7%	73.2%

ACC, adenoid cystic carcinoma area; HGT, high-grade transformation area.

EMA, epithelial membrane antigen; GCDFP-15, gross cystic disease fluid protein-15; EGFR. Epidermal growth factor receptor; AR, androgen receptor; CK, cytokeratin; ASMA, alpha-smooth muscle actin.

—, negative; f+, focally positive (1-9%); p+, partially positive (10-19%); +, positive (20-60%); ++, diffusely positive (>60%); myo, myoepithelial cells.

Finally, we diagnosed this case as “AdCC with an early phase of HGT”.

## Discussion

The tumor in this case consisted of three different components: lower-grade (cribriform subtype) of AdCC, solid-subtype AdCC and HGT. As three components existed separately, we believe that HGT occurs abruptly in typical AdCC. In the present case, HGT component lacked biphasic differentiation. Recently, Costa *et al.* reported three major criteria of AdCC-HGT [3]; 1) proliferation of tumor cells at least a focal loss of myoepithelial cells surrounding tumor nests, 2) nuclear size at least 2–3 times the size of tubular/cribriform AdCC nuclei and 3) thickened irregular nuclear membranes and prominent nucleoli in a majority of cells. The present case fulfilled at least the above 3 criteria. On the other hand, as HGT component was composed of atypical clear cells, the differential diagnosis with clear cell carcinoma, not otherwise specified (CCCNOS) or myoepithelial carcinoma is needed. However, CCCNOS is usually a low-grade malignant tumor with mildly nuclear atypia and hyalinized stroma, and is diffusely positive for p63 [4]. Therefore, “the sheet-like area” is not CCCNOS component. Clear cell type of myoepithelial carcinoma showed the positivity for myoepithelial markers, such as ASMA and p63 [5]. Therefore, “the sheet-like area” is not the component of myoepitehlial carcinoma. Moreover, the present case is different from low-grade cribriform cystadenocarcinoma (LGCCC), carcinoma ex pleomorphic adenoma (CXPA) and other rare tumors such as an epithelioid angiosarcoma. Usually, LGCCC shows the intraductal lesions with surrounding by p63-positive myoepithelial cells without atypia [6]. The present case showed cribriform pattern, but the tumor was not intraductal lesion. The criteria of CXPA are the malignant tumor, which usually consist of salivary duct carcinoma component, arising from benign pleomorphic adenoma [7]. The present case never contained the component of pleomorphic adenoma. Epithelioid angiosarcoma rarely occurs in the salivary gland, and tumor cells are infrequently positive for CK and p63 [8]. However, epithelioid angiosarcoma focally shows sinusoid-like spaces and is also positive for vimentin and CD31. The present case was negative for vimentin and CD31 (date not shown), and showed no findings such as sinusoid-like spaces.

HGT is recognized when a lower-grade malignant neoplasm abruptly transforms to a high-grade [9,10], in which the high-grade component is juxtaposed to the original lesion or the original line of differentiation is lost [1]. This concept should be distinguished from malignancy from arising a benign tumor such as CXPA, hybrid tumors, and transformation within a high-grade carcinoma to another high-grade pattern [11]. However, the gradually successive transition from solid-subtype AdCC to HGT was also reported [12].

Recently, Seethala *et al.* indicated that, in the HGT area of AdCC, the Ki-67 labeling index was >50%, and commonly showed the diffuse and strong immunopositivity of p53 protein [13]. Although Chau *et al.* indicated that, in AdCC-HGT, p53 gene mutation and overexpression of cyclin D1 and Her-2 were related to the dedifferentiation process [14], the accumulation of p53 gene product and overexpression of cyclin A, cyclin B1, and Her-2 were related to such a phenomenon in the present case. Overall, “the sheet-like area” of this tumor was high-grade carcinoma, which was considered the early phase of AdCC-HGT. The HGT component might have gene mutation of p53 and p16, and moreover, amplification of the Her-2 gene, which suggested that this component had the higher-grade malignancy. Nagao *et al.* reported 6 cases of HGT-AdCC and suggested that the HGT process was related to p53 gene mutation, overexpression of Her-2 and loss of pRb gene products [10]. In HGT of AdCC, the overexpression of p16 may be related to loss of pRb gene products, not human papilloma virus infection [15]. In the genetic analyses on AdCC with HGT, the increase of c-Myc gene and a low level increase of Her-2 gene in HGT areas were observed, but the role of Her-2 in AdCC-HGT still remains unclear [16]. However, the present case supposed that Her-2 overexpression is related to HGT at least.

Among the salivary gland carcinomas, AdCC frequently showed the HGT. Ide *et al.* reported that early phase of AdCC-HGT consisted of the scattered foci of anaplastic carcinoma in the co-/pre-existing low-grade AdCC [2]. In series of AdCC-HGT reported by Seethala *et al.*, one case had only a 20% HGT component in typical AdCC and another case contained only a 10% HGT component, who died of disease [13]. As median percentage of HGT component is 70%, which is reported by Seethela *et al.*[13], we consider that if HGT component is less than 10%, it should be included into the early phase of AdCC-HGT. The present case contained no evidence of scattered high-grade lesions, but one small focus of high-grade carcinoma existed in the central area of the AdCC. Ide *et al.* do not descript on the proportion of HGT components in the main tumor [2]. Sato *et al.* reported the gradual progression from low-/intermediate-grade AdCC to high-grade AdCC and moreover to AdCC-HGT [11]. The dedifferentiation or the HGT of the salivary gland carcinomas might occur abruptly within the co-/pre-existing low-grade carcinoma, as seen from the histological and immunohistochemical findings of the present case. In the present case, typical AdCC and HGT components were also clearly demarcated, and no findings of gradual progression were observed.

## Conclusions

In summary the early phase of the AdCC-HGT was derived from abruptly genetic and phenotypic changes. The present case is the first report that an early phase of AdCC-HGT was related to cyclin A, cyclin B1 and p16 overexpression, in addition to p53 gene product accumulation and Her-2 overexpression.

### Consent

Written informed consent was obtained from the patient for publication of this Case Report and any accompanying images.

## Abbreviations

AdCC: Adenoid cystic carcinoma; HGT: High-grade transformation; PAS: Periodic acid-Schiff; EMA: Epithelial membrane antigen; CK: Cytokeratin; CCCNOS: Clear cell carcinoma, not otherwise specified; LGCCC: Low-grade cribriform cystadenocarcinoma; CXPA: Carcinoma ex pleomorphic adenoma.

## Competing interests

The author declares to have no competing interests.

## Authors’ contributions

KK analyzed that data and wrote the manuscript as a main contributor. TM and TN conducted the pathological examination. All authors have read and approved the final manuscript.
